# Folic Acid and Birth Defects: A Case Study (Iran)

**DOI:** 10.1155/2011/370458

**Published:** 2011-12-20

**Authors:** Mohammad Bager Hosseini, Zhila Khamnian, Saeed Dastgiri, Bahram Samadi Raad, Yalda Ravanshad

**Affiliations:** ^1^Department of Neonatology, Children Health Research Centre, Tabriz University of Medical Sciences, Tabriz 515665931, Iran; ^2^Department of Community Medicine, National Public Health Management Centre (NPMC), School of Medicine, Tabriz University of Medical Sciences, Tabriz 515665931, Iran; ^3^Department of Forensic Medicine, Tabriz University of Medical Sciences, Tabriz 515665931, Iran

## Abstract

The aim of this study was to evaluate the impact of folic acid use in pregnancy for the reduction of neural tube defects (NTDs) in the northwest region of Iran. We studied 243 women with pregnancies complicated by some forms of birth defect(s). These patients were identified by medical diagnostic tests as having a fetus with some types of congenital anomalies. The prevalence of NTDs among pregnant women who were referred for therapeutic termination of pregnancy was 24.7 percent. Consumption of folic acid prevented NTDs by 79 percent (Odds Ratio = 0.21, CI 95%: 0.12–0.40) and 94 percent (Odds Ratio = 0.06, CI 95%: 0.03–0.15) compared to pregnancies complicated by other anomalies and normal pregnancies, respectively. Hydrops fetalis, hydrocephaly, Down syndrome, and limb anomalies did not have any significant association with the folic acid use. Along with the advice for the consumption of folic acid for pregnant women, they should be offered prenatal screening or diagnostic tests to identify fetal abnormalities for possible termination of pregnancy.

## 1. Introduction

At least one congenital anomaly is present in between 1% and 6% of all infants throughout the world [1]. Of all the possible birth defects, public health authorities are particularly concerned with congenital anomalies known as neural tube defects (NTDs) because they are preventable. NTDs are one of the most common birth defects in many regions [2, 3].

NTDs are now preventable with adequate intake of folic acid prior to conception and during pregnancy, which has been shown to decrease the likelihood of a child being born with a defect [4, 5].

We investigated whether preconceptional use of folic acid was associated with a reduced risk for delivering offspring with NTDs.

## 2. Methods

All pregnant women in the area are routinely examined by specialist physicians (i.e., gynecologists and obstetricians) for maternity care or possible intervention and treatment if necessary. The researchers studied 243 women with pregnancies complicated by some forms of birth defect(s) in the population under the Tabriz Registry of Congenital Anomalies (TRoCA) program. TRoCA is an established registry of birth defects covering an annual average of 20000 births in the northwest of Iran [6].

The patients were identified by medical diagnostic tests as having a fetus with some types of congenital anomalies. The patients were then referred to at least three consultant specialists for final confirmation of congenital anomalies. Following this, they were then offered termination of pregnancy according to the current national guideline for the termination of pregnancy.

Study subjects included all women referred to the clinical examination at Legal Medicine Organization (LMO) in the northwest of Iran. In Iran, it is obligatory to get permission for therapeutic abortion if a pregnancy is diagnosed as having a fetus with a major congenital anomaly. This permission is issued by LMO for eligible women. The use of folic acid and type of birth defect(s) was evaluated in those women.

We compared two groups.

Group I: 175 women who had used folic acid on a daily-based dose of 400 microgram until the end of their first trimester of pregnancy.
Group II: 68 women who never used folic acid during their pregnancies.

A group of women (*n* = 118) with normal pregnancy was also taken to assess the current situation of folic acid use in general population of pregnant women.

## 3. Results

The mean age of mothers was 28.6 years (range: 14–44 years). The majority of them (65%) had lower socioeconomic status (education and occupation).

The prevalence rate of NTDs among pregnant women who were referred for therapeutic termination of pregnancy was 24.7 percent. The occurrence of NTDs was 16.1 percent (CI 95%: 11.31–22.15) and 47.1 percent (CI 95%: 35.67–58.76) in group I and II, respectively.

Consumption of folic acid prevented NTDs by 79 percent (Odds Ratio = 0.21, CI 95%: 0.12–0.40) and 94 percent (Odds Ratio = 0.06, CI 95%: 0.03–0.15) compared to pregnancies complicated by other anomalies and normal pregnancies, respectively.

Other anomalies including hydrops fetalis, hydrocephaly, Down syndrome, and limb anomalies did not have any significant association with the use of folic acid before/during the pregnancy (Table 1).

We found that 93 percent of the general population of pregnant women with normal pregnancy take folic acid on a regular basis.

## 4. Discussion

We investigated the association between folic acid use and occurrence of NTDs in a group of women referred for therapeutic termination of pregnancy. The findings indicated that folic acid use before and after conception may have a role to reduce the rate of NTDs in the region.

Previous studies showed a prevalence rate of 7.03 (per 10,000 births in general population) for NTDs in the same area [1, 7]. We recognized/identified 60 of them in this study that referred for the termination of pregnancy because of NTDs.

Folic acid use is a routing practice in Iranian Public Health System. Ninety-three percent reported the use of folic acid in the general population of pregnant women with normal pregnancy. The same figure in women with a pregnancy complicated by a birth defect was 72 percent with an irregular use. Postma et al. reported that 49.4% of pregnant women had used folic acid to prevent fetal anomalies before pregnancy [8], whereas only 2% of Mexican pregnant women had received folic acid before pregnancy. A Cochrane Collaboration systematic review reported on the effects of increased folic acid intake through supplementation or increased consumption in the diet on NTDs and other congenital anomalies when taken periconceptionally, defined as prior to and during the first two months of pregnancy. The study population of interest was women planning to become pregnant regardless of whether they had a previous pregnancy with NTDs [9].

Our findings showed that in our region of study folic acid consumption by all pregnant women would reduce NTDs by 79 percent. This is consistent with the MRC randomized controlled trial with a reduction rate of 72 percent [10]. The results may emphasize again the necessity of folic acid fortification of grain. The flour fortification with folic acid was initiated in Iran two decades ago. However, it is still being performed as a pilot programme by the ministry of health and no formal results on the outcome have been reported yet for the whole country [11].

There were several limitations in our study: one of them was that the duration of folic acid supplementation use differed between women. The second limitation was that there was no distinction between multivitamins intended for general consumption and intended specifically for use as prenatal supplements for the prevention of birth defect(s).

## 5. Conclusion

Women in the reproductive age group should be advised about the benefits of folic acid supplementation during maternal health care visits (i.e., family planning, etc.). Along with the advice of the consumption of folic acid for pregnant women, they should also be offered prenatal screening or diagnostic tests to identify fetal abnormalities for possible termination of pregnancy. Those tests are routinely available in public and private health care sections in the country.

Since we were unable to evaluate the cost effectiveness of NTDs prevention program with folic acid, more studies are needed to assess the costs of treatment and health care services for NTD patients and the cost effectiveness of the national folic acid supplementation programs and policies. Further investigations are also recommended to investigate the etiology of NTD in the area.

## Figures and Tables

**Table 1:** Folic acid and type of anomaly.

Type of anomaly	Used		Did not use	
	*n*	% (CI 95%)	*n*	% (CI 95%)
Neural Tube Defects (NTDs)	28	16.09 (11.31–22.15)	32	47.06 (35.67–58.76)
Hydrops fetalis	20	11.49 (7.52–16.99)	6	8.82 (4.11–17.94)
Hydrocephaly	26	14.94 (10.35–20.88)	8	11.74 (6.08–21.53)
Down syndrome	16	9.19 (5.71–14.33)	5	7.35 (3.18–16.09)
Limb anomalies	20	11.49 (7.52–16.99)	2	2.94 (0.81–10.1)
Others	65	36.78 (30.33–44.51)	15	22.05 (13.85–33.26)
